# Management of sepsis and septic shock in the emergency department

**DOI:** 10.1007/s11739-021-02735-7

**Published:** 2021-04-22

**Authors:** Francesco Gavelli, Luigi Mario Castello, Gian Carlo Avanzi

**Affiliations:** 1 Department of Translational Medicine, Università del Piemonte Orientale UPO, Via Solaroli 17, Novara, Italy; 2 Emergency Medicine Department, AOU Maggiore Della Carità, Corso Mazzini 18, Novara, Italy

**Keywords:** Fluid resuscitation, Preload responsiveness, Vasopressors, Emergency medicine, Antibiotic therapy

## Abstract

Early management of sepsis and septic shock is crucial for patients’ prognosis. As the Emergency Department (ED) is the place where the first medical contact for septic patients is likely to occur, emergency physicians play an essential role in the early phases of patient management, which consists of accurate initial diagnosis, resuscitation, and early antibiotic treatment. Since the issuing of the Surviving Sepsis Campaign guidelines in 2016, several studies have been published on different aspects of sepsis management, adding a substantial amount of new information on the pathophysiology and treatment of sepsis and septic shock. In light of this emerging evidence, the present narrative review provides a comprehensive account of the recent advances in septic patient management in the ED.

## Introduction

**Fig. 1:**
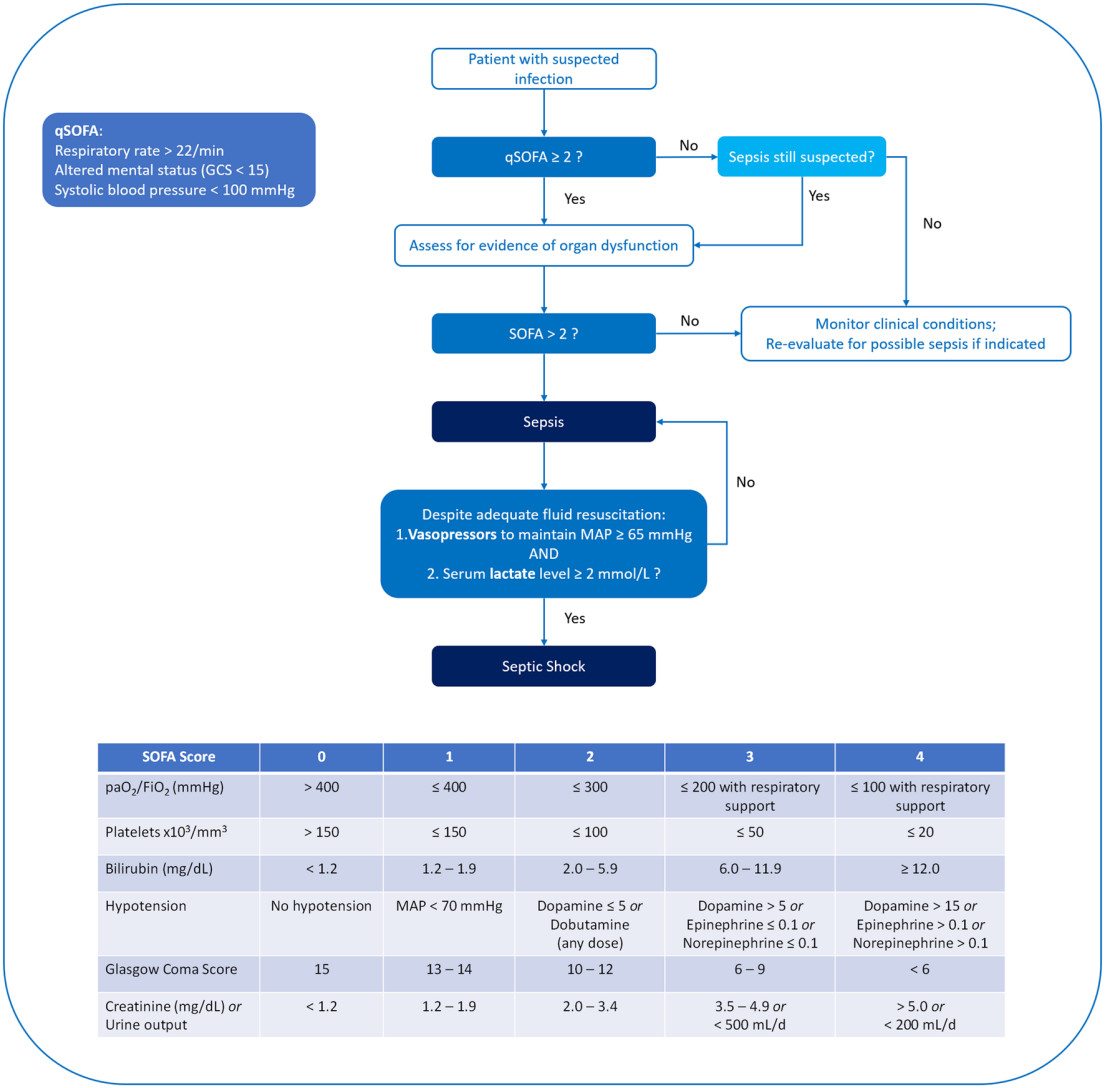
Clinical criteria for sepsis and septic shock definition. Adapted from [1]. *SOFA* Sequential Organ Failure Assessment, *qSOFA* quick-SOFA

The management of septic patients is one of the main challenges for emergency physicians. Sepsis is indeed a life-threatening organ dysfunction caused by a dysregulated host response to infection. Septic shock is a subset of sepsis, where circulatory, cellular, and metabolic abnormalities are responsible for increased mortality [1]. According to the Sepsis-3 definitions, a new algorithm involving both the Sequential Organ Failure Assessment (SOFA) and the quick-SOFA scores allows a homogeneous identification of septic patients [1] (Fig. 1). The 2016 Surviving Sepsis Campaign (SCC) [2] has provided guidelines for the management of septic patients, but the 2018 update of the SCC Bundle has stressed the need for a prompt—the hour-1 bundle—beginning of the resuscitations procedures [3].

As sepsis is a time-dependent disease, and the first medical contact of such patients takes place at the Emergency Department (ED) [4, 5], the need for early recognition and risk stratification has led to identify many prognostic markers that could help the emergency physician implement a more aggressive and effective disease management [6–10]. Nevertheless, in-hospital mortality still remains high, with rates of up to 40% in Europe and North America [11].

The aim of this narrative review is to summarize the main pathophysiological features of sepsis and septic shock, and to provide a comprehensive overview of the recent improvements in the first-line management of these conditions.

## Hemodynamic alterations

From a hemodynamic perspective, septic shock is characterized by the presence of simultaneous alterations at both the macrocirculation and microcirculation levels, resulting in an inadequate balance between oxygen demand and oxygen delivery [12].

### Derangements of macrocirculation

One of the main effects of a dysregulated inflammatory response in septic shock patients, alongside increased vascular permeability, is the depression of the vascular tone, which leads to a profound venous and arterial vasodilation [13]. Such condition is associated with a status of absolute and relative hypovolemia. The immediate clinical implication is represented by a sudden drop in arterial blood pressure, which is more evident in the diastolic component. Simultaneously, venous dilation leads to a marked reduction in the amount of stressed blood volume, thereby decreasing both venous return and cardiac output (CO), with further impairment of oxygen distribution to the tissues [14].

From a clinical perspective, the decrease in ventricular preload due to venodilation and hypovolemia is signaled by a drastic decrease in central venous pressure (CVP) [15], which triggers the activation of different neurohormonal factors aimed to maintain adequate organ perfusion, strictly dependent on the mean arterial pressure (MAP) [16]. The activation of the sympathetic tone through the stimulation of α- and β-adrenergic receptors increases both the heart rate and the cardiac contractility. At the same time, vasoconstriction induced by the stimulation of α-adrenergic receptors improves the arterial tone, which ultimately results in increased MAP. The activation of α-adrenergic receptors on veins, on the other hand, increases the venous tone, recruiting unstressed volume into stressed volume.

As the overall purpose of these series of events is to “pressurize” the circulatory system, the poor vascular response to vasoactive stimulation in septic shock patients due to adrenal insufficiency and the presence of high levels of vasodilator substances, such as oxide nitric [17], renders these compensatory mechanisms far less effective than in other forms of shock [13]. The efficacy of this compensatory response is further worsened by the impaired intrinsic contractility of both ventricles, which is observed in about 60% of septic patients [18]. Such condition, called septic cardiomyopathy, can either be present at the time of sepsis onset or appear over the following days, and seems to be related to endothelial and mitochondrial impairment, alteration of β-adrenergic receptors and myocardial calcium metabolism [18]. Even though cardiomyopathy is reversible once sepsis is resolved, it is an issue that the emergency physician should carefully take into account when treating septic patients, as it may hamper the efficacy of both compensatory mechanisms and therapeutic maneuvers.

### Derangement of microcirculation

**Fig. 2:**
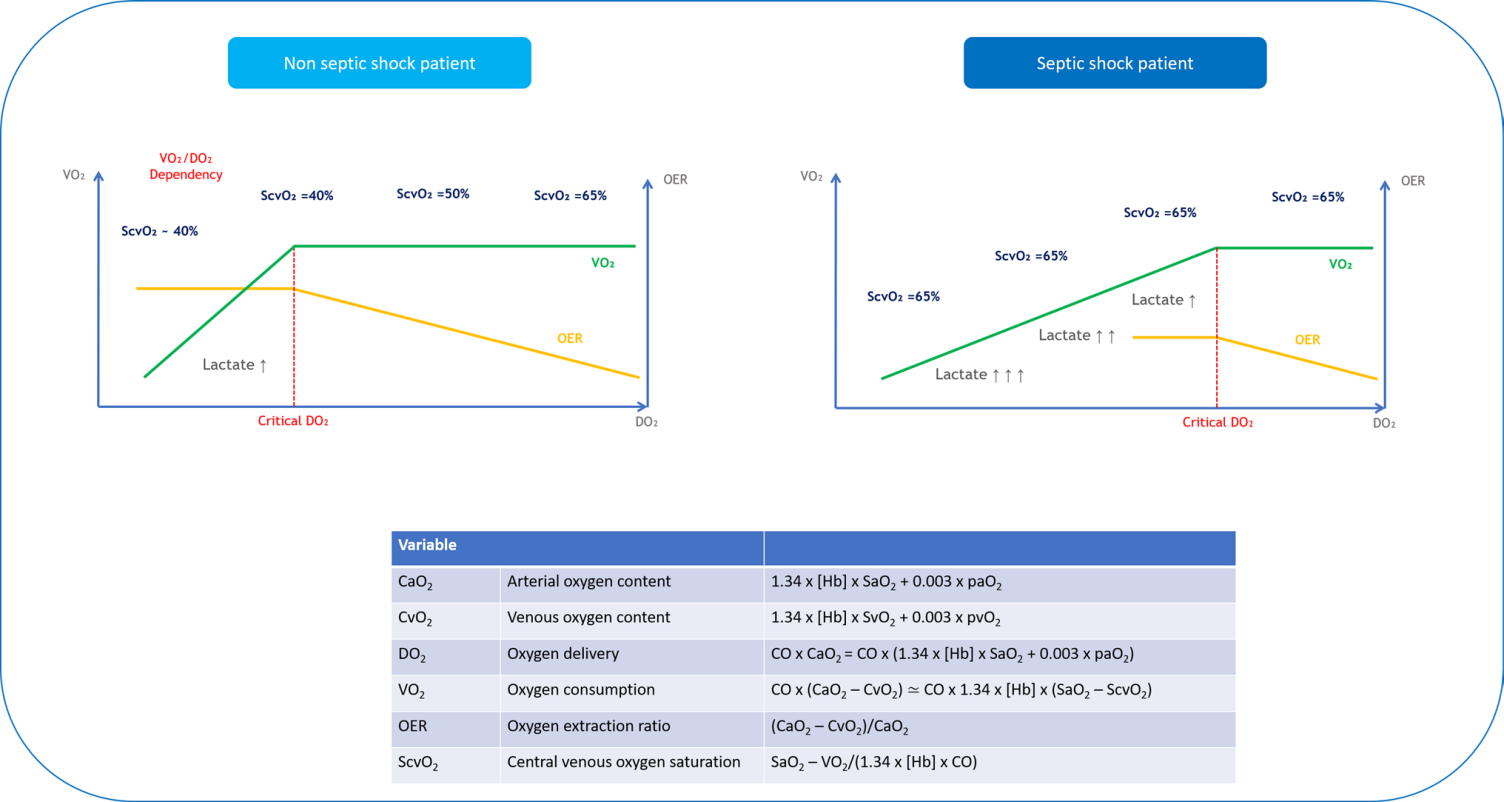
DO_2_/VO_2_ relationship in patients with or without septic shock. *CaO*_*2*_ arterial oxygen content, *CO* cardiac output, *CvO*_*2*_ venous oxygen content, *DO*_*2*_ oxygen delivery, *Hb* hemoglobin, *OER* oxygen extraction ratio, *paO*_*2*_ arterial partial pressure of oxygen, *pvO*_*2*_ venous partial pressure of oxygen, *SaO*_*2*_ arterial oxygen saturation, *ScvO*_*2*_ central venous oxygen saturation, *SvO*_*2*_ mixed venous oxygen saturation, *VO*_*2*_ oxygen consumption

Under normal conditions, oxygen delivery (DO_2_) to the tissues is higher than what is required for oxygen consumption (VO_2_), and the relationship between metabolic demands of the tissues and DO_2_ is represented by the central venous saturation (ScvO_2_) of hemoglobin [12]. In case of either increased O_2_ demand or reduced delivery, oxygen extraction increases, and this is reflected by a reduction in ScvO_2_—commonly used in place of the mixed venous saturation. However, below a certain level of DO_2_, called “critical DO_2_”, oxygen extraction cannot further increase, and ScvO_2_ cannot further decrease, making it impossible to meet the metabolic demands, which will now strictly depend on the amount of available oxygen (DO_2_/VO_2_ dependency) [12] (Fig. 2).

However, the fact that in septic shock the critical threshold of DO_2_ is higher than in any other forms of shock determines an almost immediate impairment of oxygen extraction from the tissues. It is thus very common to observe normal ScvO_2_ values in such patients [19]. The various mechanisms underlying this condition can range from microvascular derangements [20] to cellular dysoxia [21], with the latter responsible for curbing aerobic glycolysis, leading to the accumulation of lactate (Fig. 2).

## Fluid therapy

Fluid administration, together with antibiotic therapy, is the first-line therapy for septic shock patients [2]. The aim of this treatment is to correct hypovolemia by increasing the amount of stressed blood volume, with consequent increase in venous return and cardiac preload, expected to increase CO and, ultimately, oxygen delivery [14]. Nevertheless, after the initial phases of resuscitation, half of the patients will eventually become non-fluid responsive, a condition where the administration of a fluid bolus may lead to fluid accumulation, impaired DO_2_, and defective venous return, worsening the organ perfusion pressure [14]. Thus, over the years various tests have been developed to predict fluid responsiveness in septic shock patients. Among them, the passive leg raising (PLR) test has gained a lot of consensus as it is easy to perform and particularly suitable for the ED setting [22]. By moving the patient from a semi-recumbent position, lowering the trunk and raising the patient’s legs to 45°, an amount of ~ 300 mL of blood is transferred to the ventricles, thereby increasing the cardiac preload. If CO increases of at least 10% compared to baseline, the patient is considered preload responsive, thus capable of displaying a CO increase following administration of fluid. To detect the effects of a PLR test, it is recommended to adopt a method of continuous CO monitoring [23].

It is however important to point out that any septic shock patient admitted to the ED should be considered fluid responsive and immediately treated with a fluid bolus [24]. In this regard, the 2016 SCC guidelines, recommending a fixed dose of 30 mL/kg of crystalloids within the first 3 h [2], sparked an intense debate among clinicians. On the one hand, the timeframe of 3 h before patient revaluation seemed too long for a time-dependent disease such as septic shock. On the other hand, the recommended amount of fluid was deemed too liberal, hardly suitable for all patients [25]. These concerns were further justified by the lack of high-quality data supporting these recommendations.

The 2018 SCC bundle update partially resolved this controversy by switching from the previously recommended 3- and 6 h bundles to an hour-1 bundle, recognizing the need for an immediate treatment of septic patients [3]. Nonetheless, no changes to the protocol were made to personalize fluid administration, as advocated by many. In this regard, Teboul and Monnet have recently proposed to start fluid administration with an infusion of ~ 10 mL/kg within the first 30 to 60 min while closely monitoring the patient [25]. In case of worsening of tachypnea or a drop in oxygen saturation, the amount of fluid should be reduced. Conversely, an increase in the infusion rate should be considered if signs of low arterial pulse pressure, increased capillary refill time, or skin mottling were to persist despite initial fluid treatment. We fully agree with this approach because we feel that the decision to continue fluid administration should be based on the individualized risk/benefit ratio of the patient, using dynamic tests of preload responsiveness to evaluate the benefit of further fluid infusion.

### What kind of fluid should the patient be given?

The 2016 SCC guidelines suggest the use of crystalloids for fluid resuscitation of septic patients [2], but over the years different types of fluids have been proposed. Among them, the use of synthetic colloids, such as hydroxyethyl-starch solutions (HES), was based on the assumption that their intravascular volume expansion would have been superior to that of crystalloids [26]. After the publication of several studies indicating that HES treatment was associated with increased renal damage and mortality [27, 28] and the retraction of many articles supporting the use of colloids in sepsis [29], in 2013 the European Medicines Agency (EMA) banned their use for the treatment of septic and critically ill patients [30]. Of note, in a retrospective cohort study it was shown that the volume of crystalloids given for fluid resuscitation in critically ill patients was on average only 1.4 times higher than that of colloids [31]. Currently, the only indication for HES remains the treatment of hypovolemia due to acute blood loss when crystalloids alone are not considered sufficient [30].

Regarding the choice of which isotonic crystalloid should be administer—i.e., balanced crystalloids *vs*. normal saline solution—over the years no clear recommendations have been made. On the one hand, it has often been advocated that saline solution could exert detrimental effects on both renal function and the patient’s ability to recover from severe illnesses, due to its large amount of chloride [32]. On the other hand, no study has been able to demonstrate a clear superiority of balanced crystalloids over saline solution in critically ill patients. In this regard, in non-critically ill patients treated in the ED with saline or balanced crystalloids, the SALT-ED study confirmed no differences in terms of in-hospital free days [33]. Nonetheless, the authors demonstrated a significant lower incidence of major kidney adverse events within 30 days in the balanced crystalloids group. In contrast, the SMART trial, which compared the efficacy of the two solutions in a cohort of 15,802 critically ill patients, reported a lower rate of the composite outcome of death from any cause, new renal-replacement therapy, or persistent renal dysfunction when balanced crystalloids were used [34]. The secondary analysis conducted on the subgroup of septic patients (*n* = 1641) also demonstrated a lower 30 day in-hospital mortality, as well as a lower incidence of major renal events and a higher number of vasopressor-free days when balanced crystalloids were given compared to normal saline. Therefore, even though no clear recommendations have been made from the Scientific Societies since then, the administration of Ringer Lactate solution in the resuscitation of septic shock patients may be considered appropriate. Although in the SMART trial [34] the septic subgroup was pre-specified, and the number of patients afforded a great statistical power, the results of the two ongoing PLUS (Plasma-Lyte 148 *vs* Saline) [35] and BaSICS (Balanced Solution *vs* Saline in Intensive Care) [36] studies are expected to confirm such findings.

Lastly, the use of a hypertonic solution in resuscitation of septic shock patients has been recently put into question following the premature stop of the HYPERS2S trial, evaluating both hyperoxia *vs.* normoxia and normal saline *vs.* hypertonic saline, due to increased mortality in both intervention groups [37]. Even though the rationale behind the administration of a hypertonic solution (i.e., 3% NaCl) to septic shock patients is that this treatment should exert a greater osmotic effect, such effect does not often last very long, making the hypertonic effect futile. In addition, it consists of the administration of a very large amount of chloride, which increases the risk of renal damage. For this reason, we believe that infusing septic shock patients with a hypertonic saline solution should not be recommended in the daily clinical practice.

## Vasopressors

According to the Sepsis-3 definitions, septic shock patients can be clinically identified by the requirement of vasoactive medicaments (Fig. 1) [1]. In this regard, both the 2016 SSC guidelines and the 2018 SSC bundle recommend the early administration of vasopressors in hypotensive septic patients to revert the severely impaired arterial tone [2, 3].

### Norepinephrine

Norepinephrine (NE) is recommended as the first-line vasoactive agent in the management of septic shock patients [2]. Its vasoconstrictive action is exerted through the stimulation of α1-adrenergic receptors, with little influence on the heart rate [38].

Over the years, there has been a growing consensus for the need of early NE administration in septic patients, supported by a series of validated reasons [39]. The first and most obvious one is that, by reversing hypotension or limiting its duration—prolonged hypotension is among the main determinants of mortality—patient outcome is improved. Another reason supporting early NE administration is that the stimulation of α1-receptors on the venous side triggers venous constriction and increases the amount of stressed blood volume [40]. This leads to enhanced venous return and improved cardiac preload. Of note, fluid administration under such conditions should be more efficient as it would be performed in a more pressurized venous system, thus acting on the stressed volume and, ultimately, reducing the amount of fluid given [41]. Lastly, the fact that in the initial phases of septic shock cardiac β1-adrenergic receptors are still expressed on cardiac cells allows cardiac contractility to increase through NE administration [42]. This beneficial effect of NE is also promoted by a concomitant increase in diastolic arterial pressure, which is the perfusion pressure of the left ventricle coronary artery [42].

Different studies have evaluated the effects of early NE administration in septic shock patients. Two retrospective studies by Colon-Hidalgo [43] and Bai [44] have shown that the time to NE initiation is an independent predictor of mortality. In this regard the CENSER trial has directly compared early NE administration to NE administration only when fluid therapy has failed [45]. The results of this trial have shown that early NE administration is associated with increased shock control over the first 6 h (primary endpoint). However, no significant differences in terms of mortality were observed (secondary endpoint), even though the early NE group had significant lower rates of cardiogenic pulmonary oedema and new-onset arrhythmias [45]. In this regard, a large ongoing randomized controlled trial (RCT) (the CLOVERS trial), specifically designed to evaluate the effects of early NE administration on 90-day mortality, is expected to provide more definitive results on this issue (ClinicalTrials.gov Identifier: NCT03434028).

However, the existing data, along with those of a recent meta-analysis showing a significant reduction in both short-term mortality and the amount of fluid given, suggest that the early administration of NE is safe [46]. Thus, the emergency physician should be encouraged to start NE administration right from the initial phases of resuscitation, especially in case of concurrent tachycardia and low diastolic arterial pressure, which is a sign of strongly impaired arterial tone [47].

Once NE administration is started, the consensus is that its dosage should be titrated to obtain a MAP of 65 mmHg [2]. It is not however clear whether higher values should be targeted. In this regard, the SEPSISPAM study, which compared 65 mmHg *vs*. 85 mmHg as MAP target, did not find significant differences in terms of mortality [48]. Nevertheless, when the subgroup of patients with a history of arterial hypertension was analysed, a higher MAP target exerted a beneficial effect on the renal function. Thus, a task force of the European Society of Intensive Care Medicine (ESICM) has recommended that a MAP value greater than 65 mmHg should be the initial blood pressure target in septic shock patients with arterial hypertension [49]. When elevated doses of NE (≥ 1 µg/Kg/min) are required for refractory hypotension, the use a second vasopressor is advised.

### Other vasoactive agents

Vasopressin is the vasoactive agent that the 2016 SSC guidelines suggest to add to NE in case of refractory shock [2] to reduce the amount of adrenergic tone and increase vasoconstriction through a different receptor stimulation. In this regard, one meta-analysis showed that when vasopressin was associated to NE, the rate of arrhythmic events, such as atrial fibrillation, was reduced compared to NE alone, but no differences on mortality were recorded [50]. It is however important to point out that vasopressin is not available in all countries.

Epinephrine is another second-line vasopressor recommended by the 2016 SSC, whose use should be considered in case of concurrent cardiac dysfunction [2]. However, according to the existing literature, no superiority in terms of patient survival was observed in patients treated with epinephrine alone compared to patients treated with a combination of NE and dobutamine.

Dopamine, which was recommended by previous guidelines, should not be used in the management of septic patients as either vasopressor or, at low doses, renal protective agent [2]. It has been shown that its use is associated with an increased risk of cardiac arrhythmias and mortality, compared to NE [51]. Currently, its use is only recommended in case of bradycardia.

## Monitoring

Despite its intrinsic limitations, physical examination is widely regarded as an essential tool for septic shock recognition and initial disease management, [52]. Likewise, basic monitoring, such as heart rate, peripheral oxygen saturation, urinary output, arterial blood pressure, and CVP, can provide important information on the hemodynamic status of septic shock patients. These easily obtainable variables allow the physician to not only detect rapid changes but also identify specific targets for resuscitation (e.g., MAP ≥ 65 mmHg) [47, 53].

In case of vasopressors administration, invasive arterial blood pressure monitoring is suggested, albeit neither arterial catheter nor central venous catheter (CVC) placement should delay NE administration. Under these conditions, vasopressor treatment can be initiated on a peripheral venous line with non-invasive BP monitoring, and it should be shifted, as soon as possible, to CVC administration accompanied by invasive arterial pressure monitoring [41, 54]. As basic hemodynamic monitoring is not able to detect the effects of fluid challenge on CO [55, 56], methods for continuous CO monitoring are recommended to track changes in CO both during tests of preload responsiveness [22, 57, 58] and fluid challenge [23, 59].

### Minimally invasive and non-invasive monitoring methods

Over the last two decades, the use of the pulmonary artery catheter has sharply declined, making transpulmonary thermodilution (TPTD) the new gold standard techniques for CO measurement. Through the injection of 3 boluses of cold saline in the CVC and the detection of changes in blood temperature by means of a femoral thermistor-tipped arterial catheter, the TPTD device can measure CO [60]. However, its use in septic patients is recommended in the presence of acute respiratory distress syndrome in the ICU rather than the ED [49, 61].

Thus, various non-invasive methods for CO monitoring have been developed over the years. Among them, the analysis of the arterial pulse wave contour, which is proportional to CO, is a method that requires the placement of an arterial line with a dedicated catheter [62]. CO is estimated through proprietary algorithms at each cardiac beat, indexed to biometric parameters. However, since it does not have a calibrating system—conversely to TPTD devices—after some time from its placement, or in case of changes in vascular resistance, its measurements become less reliable [52].

Echocardiography is, on the other hand, a completely non-invasive and rapidly available tool at the bedside. CO can be assessed by evaluating relative changes in the velocity–time integral (VTI) of the left ventricular outflow tract [63]. This technique allows the evaluation of beat-to-beat changes in CO during both tests of preload responsiveness and fluid administration, with good accuracy and inter-observer concordance [63]. Finally, in recent years different new techniques have been developed, improved, and validated for non-invasive CO monitoring, ranging from bioreactance [64] to plethysmography [65, 66], which may be optimal for the ED setting. It should be noticed that inferior vena cava variations must not be used to assess preload responsiveness or response to fluid administration in spontaneously breathing patients [67].

### Lactate and peripheral perfusion assessment

According to the 2016 SSC guidelines, blood lactate normalization is one of the target of resuscitation in septic shock patients [2], supposing that high lactate levels reflect the degree of tissue hypoperfusion. However, the fact that lactate levels depend on the balance between lactate production and clearance may slow down the kinetics of blood lactate normalization in resuscitating patients. This could be further complicated by the possibility that hyperlactatemia may be related to causes other than hypoperfusion [68]. In this regard, capillary refill time (CRT), defined as the time taken for a distal capillary bed to regain its color after pressure has been applied to cause blanching, has emerged over the recent years as a tool for the assessment of peripheral tissue perfusion [69]. In 2019, the ANDROMEDA-SHOCK study [70] compared peripheral perfusion-targeted *vs*. lactate-targeted resuscitation in terms of mortality in 424 septic shock patients. Even though it failed to reach statistical significance (*p* = 0.06), the CRT-targeted strategy was associated to lower mortality at 28 days, which has been recently confirmed by several *post-hoc* analyses [71–73].

## Antimicrobial therapy

Antimicrobial therapy, together with fluid resuscitation, is the cornerstone of septic patients treatment [2, 3]. Provided that it does not determine substantial delays in the initiation of the treatment, antibiotic administration should be preceded by appropriate routine microbiological cultures [2]. According to guidelines, two sets of blood cultures must always be collected, notably one for aerobics and one for anaerobics.

### When should the antimicrobial therapy be started?

The 2016 SSC guidelines recommend that intravenous antibiotic administration should begin within one hour after sepsis and septic shock recognition [2]. In this regard, many studies have highlighted the detrimental role of late antibiotic administration in septic patients [74–77]. Among the most recent ones, Liu et al*.* [78] performed a retrospective analysis of 35,000 septic patients from 21 ED in the US, for whom antibiotic therapy was administered within 6 h of arrival. In their analysis, they demonstrated that a delay in antibiotic administration significantly increased the adjusted in-hospital mortality with an odds ratio (OR) of 1.09 (1.05–1.13) for each hour of delay. In the unadjusted analysis, the subgroup of septic shock patients emerged to be the one in which such effect was more pronounced [78]. Similarly, on a retrospective analysis of 40,696 septic and septic shock patients, Seymour et al*.* [79] demonstrated that an increased time to antibiotic administration was associated with a higher risk-adjusted in-hospital mortality [OR 1.04 (1.03–1.06) per hour]. More recently, Kashouris et al*.* evaluated the impact of the time between prescription and antimicrobial administration on the mortality rate among a cohort of 4429 septic patients. The authors showed that the OR for 28 day mortality increased when antimicrobial therapy was administered beyond 1 h, reaching a median value of 1.85 (1.29–2.65) if the delay was more than 12 h [80]. Likewise, a retrospective analysis of 10,811 ED septic patients showed that each additional hour from ED arrival to antibiotic initiation increased the odds of 1 year [1.10 (1.05–1.14)], in-hospital [1.16 (1.07–1.26)], 30 day [1.12 (1.06–1.18)] and 90 day [1.09 (1.04–1.15)] mortality [81].

### What antimicrobial therapy should I start with?

As the time to the first dose of antibiotic is crucial, an empiric broad-spectrum antimicrobial therapy must be initiated as soon as possible, until the underlying pathogen is recognized and antimicrobial sensitivities are established [2]. In this regard, the initial choice should consider different issues, such as the primary site of infection, the prevalent pathogens and antimicrobial resistances in that geographical area, and the patient’s age and comorbidities. However, due to the variety of such conditions in the population of septic patients, the 2016 SSC could not issue specific therapeutic regimens, but rather a series of suggestions [2].

Thus, as most of the septic shock patients exhibit a various degree of immunosuppression, the initial treatment should target pathogens frequently encountered in healthcare-associated infections, notably Gram-negative pathogens. The SSC guidelines suggest initiating with either a broad-spectrum carbapenem (e.g., meropenem, imipenem/cilastatin) or an extended-range penicillin/β-lactamase inhibitor (e.g., piperacillin/tazobactam, ticarcillin/clavulanate), even though third or higher-generation cephalosporins may also be used. However, to be more effective, the SSC guidelines adopt the concept of “multidrug therapy”, suggesting that a combination of multiple antimicrobials may be more effective for broad spectrum coverage [2]. As a matter of fact, in critically ill patients at high risk of infection from multidrug-resistant pathogens, it is recommended to add a supplemental Gram-negative agent (e.g., aminoglycoside, fluoroquinolone) to improve the probability of having at least one efficient antibiotic [82]. Similarly, in case of suspected MRSA-related sepsis, it is recommended to add either vancomycin, teicoplanin, or another anti-MRSA agent. Again, in the presence of patients at high risk for invasive *Candida* infection, the empiric addition of an echinocandin (e.g., caspofungin, anidulafungin, and micafungin) is regarded as a reasonable choice [2]. In case of doubts, the clinician should seek consultation with an infectious disease specialist.

Once the pathogen has been identified and antimicrobial sensitivities have been determined, the broad-spectrum treatment should be discontinued and a targeted/definitive therapy, either mono- or combination, initiated [2, 82].

### What antimicrobial dose should be used in the ED?

The emergency physician should always bear in mind the hemodynamic alterations of septic shock when prescribing the first administration of antibiotic, since recommended doses are often inadequate to reach the therapeutic target. As a matter of fact, the increased capillary permeability, the hyperdynamic state, and the large amount of fluid administered may all contribute to increase the volume of distribution of the medication, especially for hydrophilic antibiotics (e.g., β-lactams, aminoglycosides, glycopeptides) [83]. Similarly, for highly protein-bound antimicrobials, such as, ceftriaxone, ertapenem, daptomycin, and teicoplanin, the presence of hypoalbuminemia may augment the unbound fraction, increasing the volume of distribution and the risk of early renal clearance [84]. For these reasons, it is recommended to increase the loading dose of roughly 1.5 times the standard dose [13, 85, 86]. However, to optimize the efficacy of antimicrobial therapy, also the pharmacokinetic/pharmacodynamic (PK-PD) index for each class of antibiotic should be taken into account, especially for maintenance doses (e.g., shortening dosing intervals, continuous infusions, etc.) [87, 88] (Table 1). Nevertheless, even in this case, due to the pathophysiological complexity of the septic shock it is difficult to predict the response to an antimicrobial regimen. Therefore, the implementation of antimicrobial therapeutic drug monitoring has been suggested as standard of care in septic shock patients treated with different types of antibiotics [89].

**Table 1:** Pharmacokinetic/Pharmacodynamic indices of different antimicrobial classes and suggestions for dose adjustment in critically ill patients

Antimicrobial class	PK/PD index	Clinical PK/PD target for efficacy	Clinical PK/PD threshold for toxicity	Adjustment in critically ill patients
Aminoglycosides	AUC_0-24_/MIC and C_max_/MIC			High, single dose and extended interval dosing
Amikacin		C_max_/MIC ≥ 8–10	C_min_ > 5 mg/L	
Gentamicin/Tobramycin		AUC_0-24_/MIC ≥ 110 C_max_/MIC ≥ 8–10	C_min_ > 1 mg/L	
Beta-lactams	% fT_>MIC_			Initial loading dose, followed by prolonged (continuous or extended) infusion
Carbapenems		50–100% fT_>MIC_	C_min_ > 44.5 mg/L	
Cephalosporin		45–100% fT_>MIC_	C_min_ > 20 mg/L	
Penicillins		50–100% fT_>MIC_	C_min_ > 361 mg/L	
Daptomycin	AUC_0-24_/MIC	AUC_0-24_/MIC ≥ 666 mg/L	C_min_ > 24 mg/L	Higher doses (10–12 mg/kg/day) to increase MIC (> 0.1 mg/L)
Fluoroquinolones	AUC_0-24_/MIC and C_max_/MIC	AUC_0-24_/MIC ≥ 125–250 C_max_/MIC ≥ 12	Unclear	Loading dose with higher maintenance doses should be considered
Glycopeptides	AUC_0-24_/MIC			
Teicoplanin		C_min_ ≥ 10 mg/L	Unclear	Loading dose essential to reduce time to reach therapeutic exposures
Vancomycin		AUC_0-24_/MIC ≥ 400 C_min_ > 10–20 mg/L	AUC_0-24_ > 700 C_min_ > 20 mg/L	Consider loading dose of 25–30 mg/kg, followed by 15–20 mg/kg every 8–12 h if MIC > 1 mg/L and no renal impairment
Linezolid		AUC_0-24_/MIC 80–120 ≥ 85% T_>MIC_	AUC_0-24_ > 300–350 C_min_ > 7–10	Higher doses can be considered in ARDS and obese patients, or if MIC ≥ 2 mg/L
Echinocandins	AUC_0-24_/MIC	AUC_0-24_/MIC ≥ 3000	No data	Higher body weight may require higher dosing
Fluconazole	AUC_0-24_/MIC	AUC_0-24_/MIC ≥ 55–100	Unclear	Loading dose of 12 mg/kg IV, followed by 6–12 mg/kg/day to reach therapeutic targets (AUC_0-24_/MIC 25–100), if no renal impairment
Voriconazole	AUC_0-24_/MIC	C_min_ ≥ 1–2 mg/L	C_min_ ≥ 4.5–6 mg/L	Loading dose of 6 mg/kg IV every 12 h for two doses, followed by 3–4 mg/kg IV every 12 h

Adapted from [89]

*AUC*_*0-24*_*/MIC* ratio of the area under the concentration–time curve over a 24 h period to minimum inhibitory concentration, *C*_*max*_*/MIC* ratio of the maximum drug concentration to minimum inhibitory concentration, *C*_*min*_ minimum drug concentration, *fT*_*>MIC*_ duration of time that the free drug concentration remains above the minimum inhibitory concentration during a dosing interval, *IV* intravenously, *MIC* minimum inhibitory concentration, *PK/PD* pharmacokinetic/pharmacodynamic

## Adjunctive therapy

### Steroids

The presence of a relative adrenal insufficiency is the main rationale for the administration of low-dose steroids in septic shock patients [90]. In theory, steroids are expected to improve the cardiovascular function by not just restoring the blood volume through mineralocorticoid activity but also increasing the systemic vascular resistance, which is partially mediated by glucocorticoid receptors [91]. In 2008, the CORTICUS trial evaluated the effects of IV administration of 50 mg hydrocortisone every 6 h for five days, compared to placebo, in 499 septic shock patients [92]. The results of this large multicenter RCT showed no difference in terms of 28-day mortality between patients who did not have an appropriate response to a corticotropin test (primary outcome) and patients who instead were responsive to such test [92]. It was however observed a quicker reversal of shock in the hydrocortisone group, together with increased risk of developing superinfections. Since then, controversial results have been reported by different reviews and meta-analyses. Among them, Annane et al*.* [93], analyzing 12 clinical trials, showed that prolonged low-dose corticosteroid therapy reduced 28-day mortality, whereas Sligl et al*.* [94] and Volbeda et al*.* [95] could not reach the same conclusions examining 8 and 35 trials, respectively. Thus, both the 2012 and 2016 SSC guidelines have recommended the use of low-dose corticosteroids (notably hydrocortisone 200 mg IV once a day) only in patients with severe shock, unresponsive to fluids and vasopressors [2, 96].

In 2018, the results from two large RCTs were published, namely ADRENAL and APROCCHSS [91, 97]. The former enrolled 3658 patients with septic shock under mechanical ventilation and measured as primary outcome the differences in mortality at 90 days between patients treated with hydrocortisone 200 mg IV once a day for seven days and the placebo group. Even though the results showed a faster resolution of shock, fewer days on mechanical ventilation, and shorter ICU length of stay in the hydrocortisone group, no differences were observed in terms of 90-day mortality [97]. On the other hand, the APROCCHSS trial [91] enrolled 1241 septic shock patients in whom clinical conditions did not improve after initial resuscitation, according to the 6 h bundle of the 2008 SSC [98]. Patients were randomly assigned to receive either hydrocortisone (50 mg IV every 6 h) *plus* fludrocortisone (50 µg tablet once/day) for seven days without tapering, or placebo. Compared to the placebo group, the hydrocortisone *plus* fludrocortisone-treated group displayed a substantial decrease in mortality at 90 (primary outcome) and 180 days, as well as a significant increase in the number of vasopressor- and organ failure-free days. The authors stated that fludrocortisone had been added to hydrocortisone to provide additional mineralocorticoid potency, following the assumption that the increased mineralocorticoid activity would have counteracted NF-κB-mediated downregulation of vascular mineralocorticoid receptors, further improving the cardiovascular function [91]. As the systematic reviews and meta-analyses including these two large trials provided once again conflicting results [99, 100], the recommendations issued by the 2016 SSC on this issue still hold true [2].

### Ascorbic acid and thiamine

The therapeutic role of vitamin C in septic patients has been proposed by Marik et al*.* in a small-size retrospective study, where it was shown that intravenous administration of this vitamin in combination with thiamine and hydrocortisone (HAT therapy) reduced both mortality and organ failure in sepsis and septic shock patients [101]. This improved patient outcome could be the result of the synergistic and overlapping actions on different components of the host immune response to infection, such as restoration of the dysregulated immune system [102]. Since then, different small-size RCTs have investigated the efficacy of HAT therapy in sepsis and septic shock patients [103–106] with discordant results. In this regard, a meta-analysis by Rui Shi et al*.* [107] did not report a clear benefit on mortality in HAT-treated patients, whereas it was recorded a significant reduction in vasopressor administration frequency as well as a decreased SOFA score [107]. The results of the ongoing VICTAS Trial [108], which is expected to enroll 2000 septic patients, may lead to more robust conclusions.

## Conclusions

Patients with severe sepsis and/or septic shock are at increased risk of death and organ dysfunction and display high in-hospital mortality. Since the last SSC guidelines were issued, a number of studies have provided new information on the pathophysiology and treatment of septic shock. Despite this growing knowledge, septic shock management remains a challenging task for the emergency physicians, who have to deal with the initial detection of the condition and the early phases of treatment. Thus, it is of utmost importance that emergency physicians be aware of the recent advances on septic patient management.

In light of the above considerations, this narrative review provides a useful and updated learning tool that should enable emergency physicians to gather crucial information on past, present, and future research trajectories of sepsis research.

Overall, we believe that a systematic approach consisting of coordinated detection of patients with sepsis and early treatment could significantly reduce the mortality of septic patients in the ED. To further optimize disease management, the emergency physician should administer treatment taking into account the patients’ characteristics.
